# Supplementary figures and images for: Single-Cell RNA Sequencing Reveals an Atlas of Meihua Pig Testis Cells
Source: Animals (Basel). 2025 Mar 5;15(5):752. doi: 10.3390/ani15050752 (PMC11899385; doi:10.3390/ani15050752)

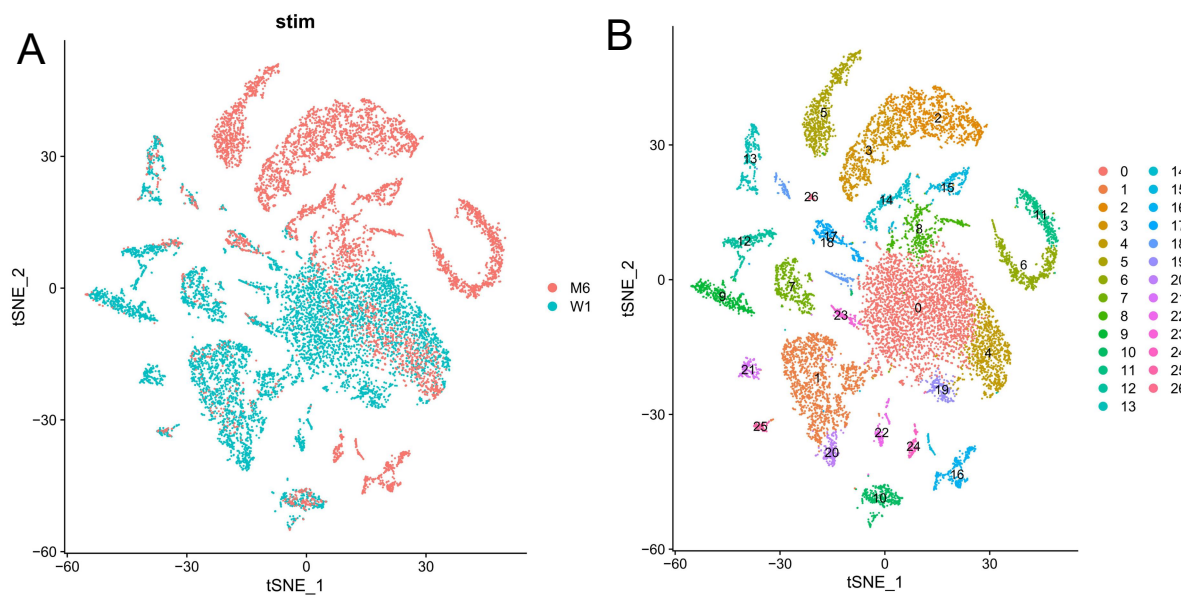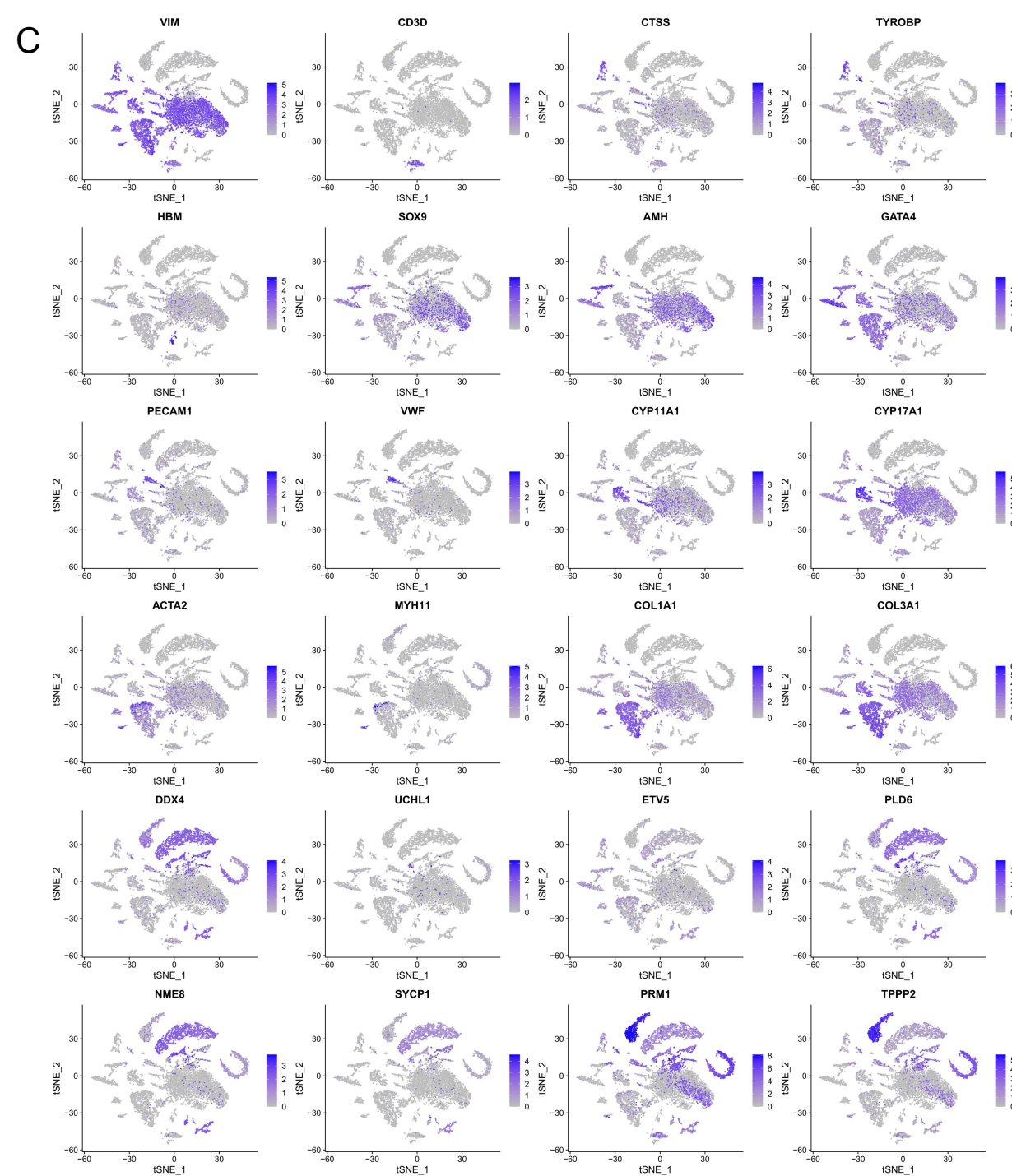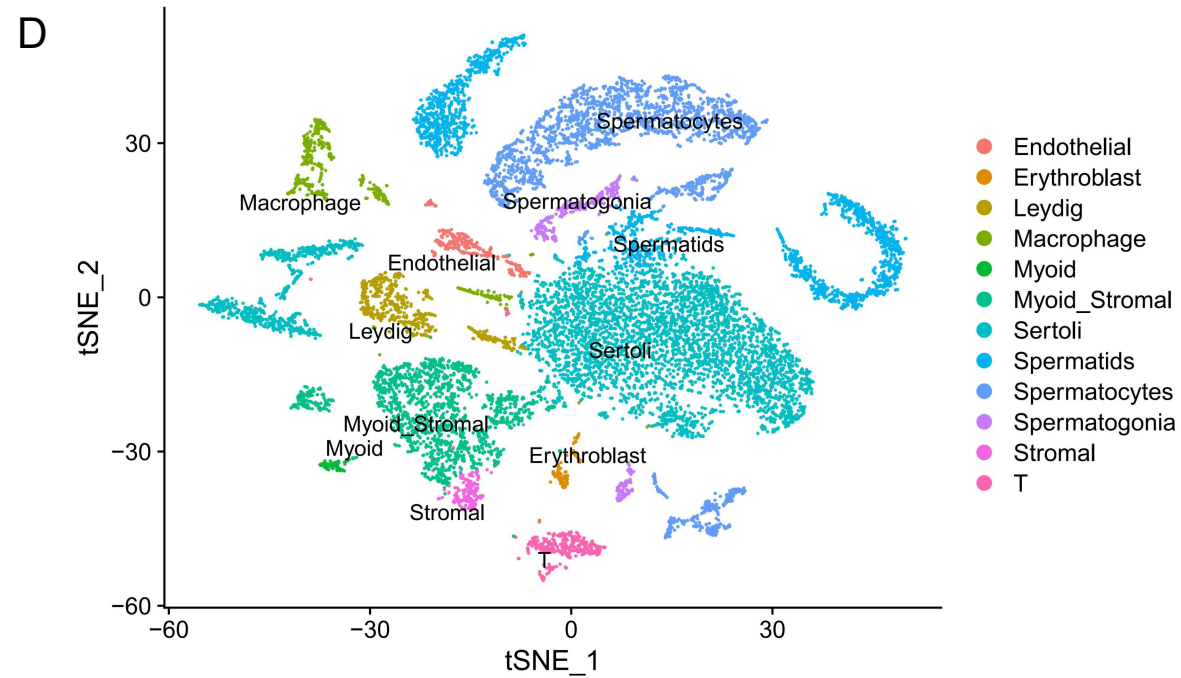

Supplement: Supplementary file 1 [file animals-15-00752-s001.zip › Supplementary File/Figure S1.pdf]

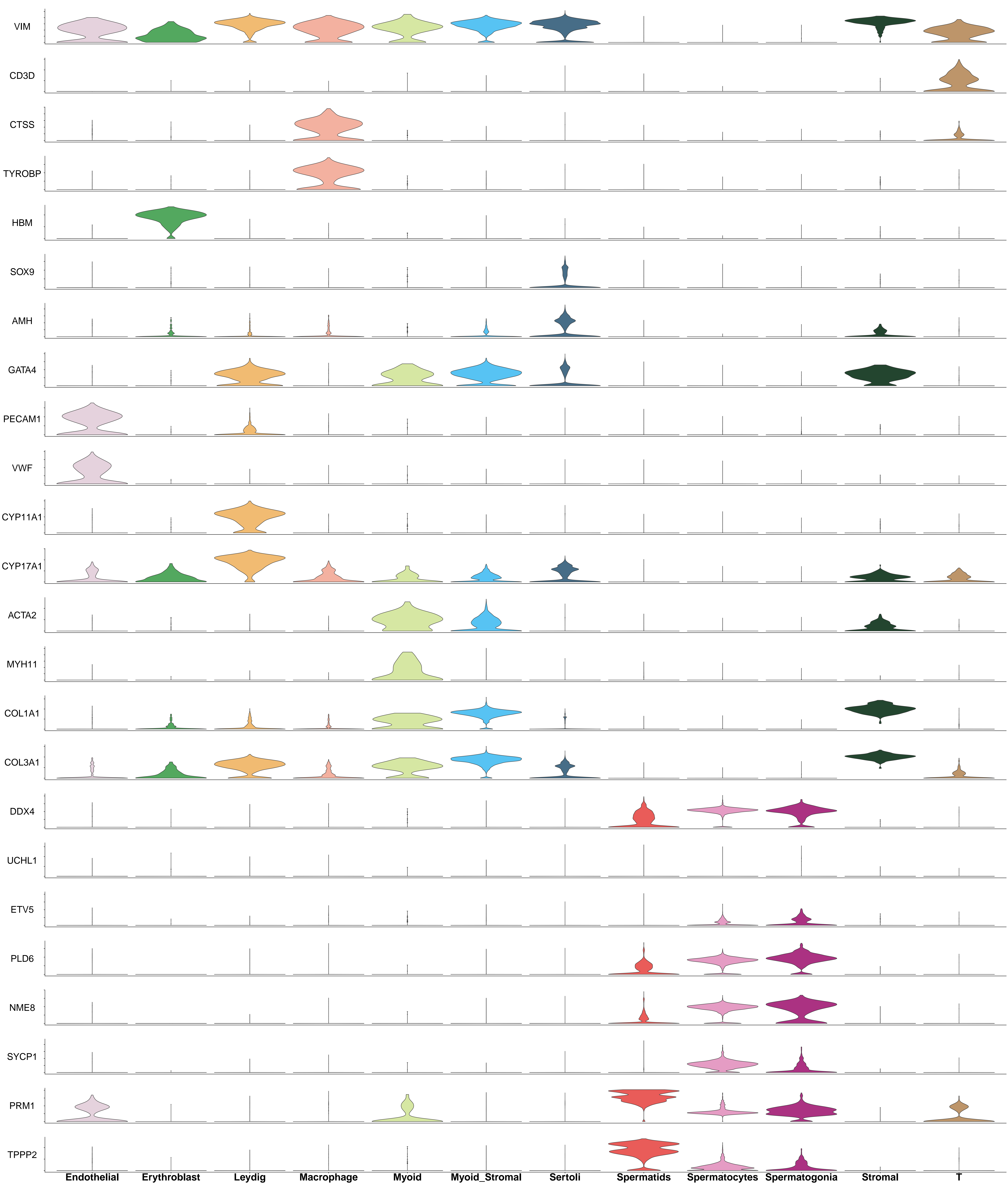

Supplement: Supplementary file 1 [file animals-15-00752-s001.zip › Supplementary File/Figure S2.pdf]

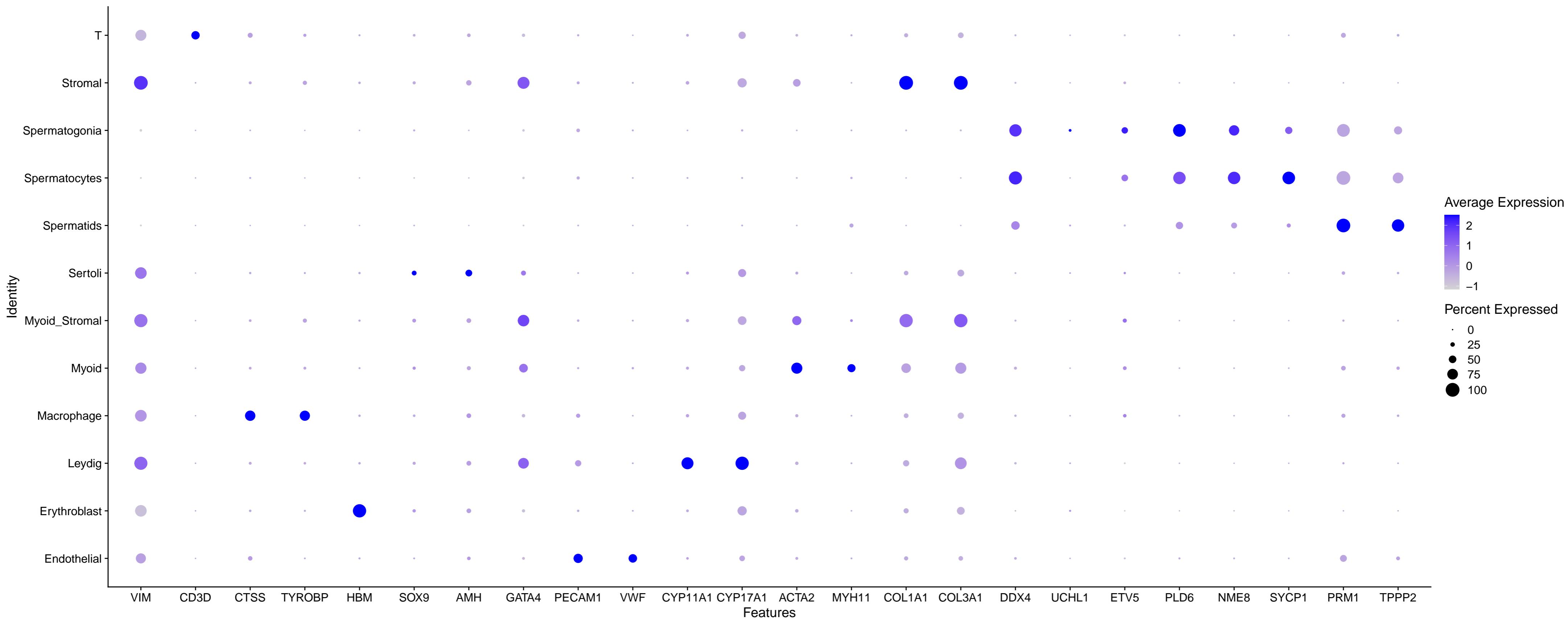

Supplement: Supplementary file 1 [file animals-15-00752-s001.zip › Supplementary File/Figure S3.pdf]

A

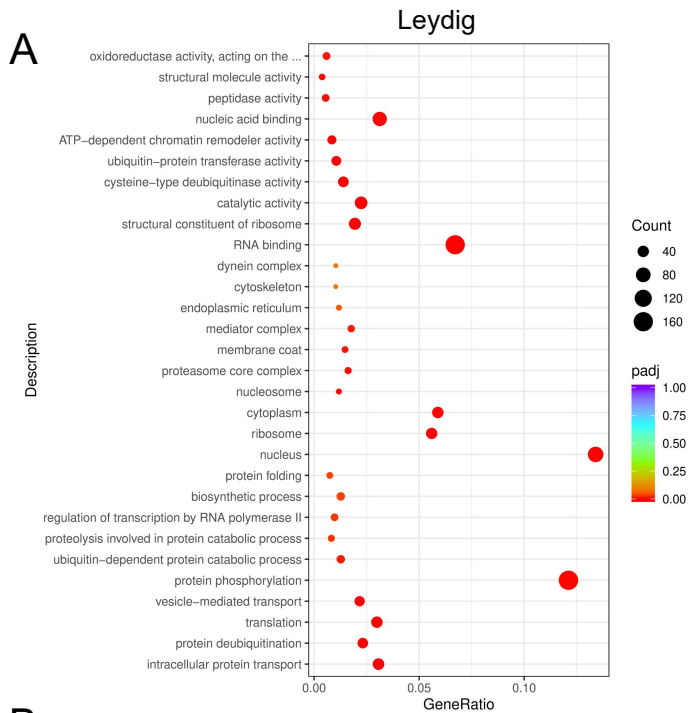

Endothelial

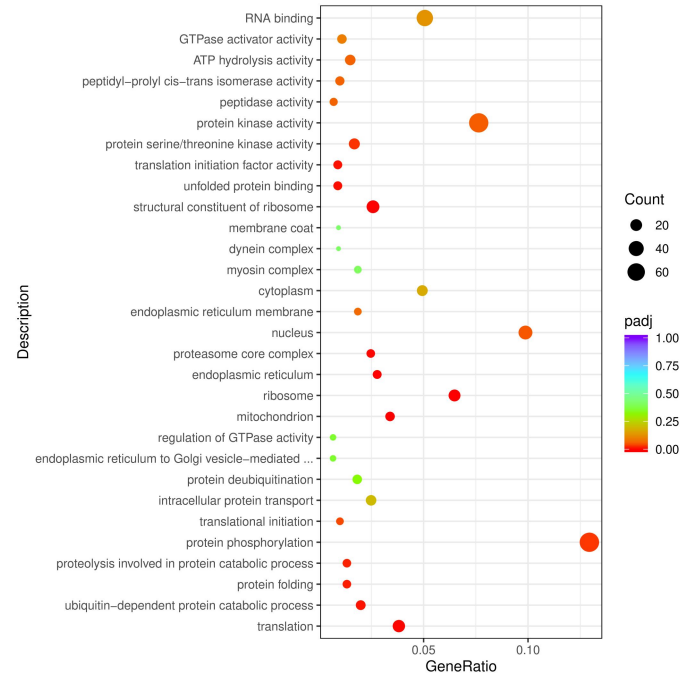

T/macrophage

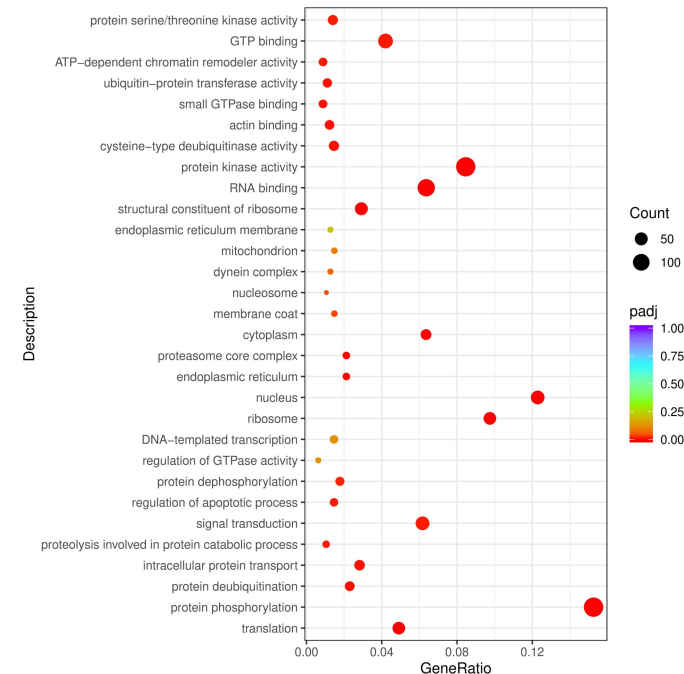

B

Leydig

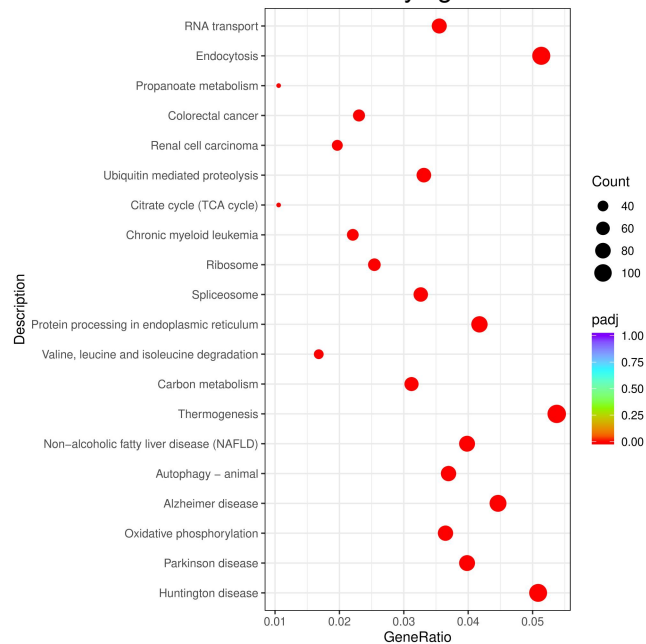

Endothelial

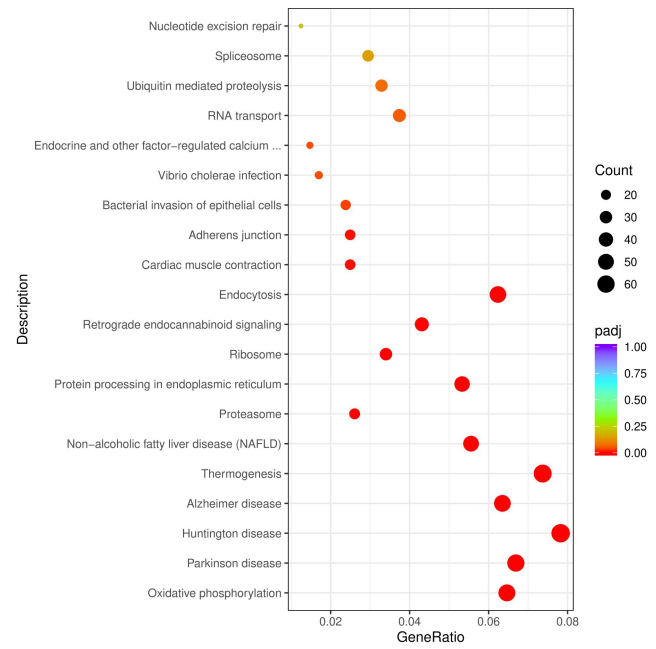

T/macrophage

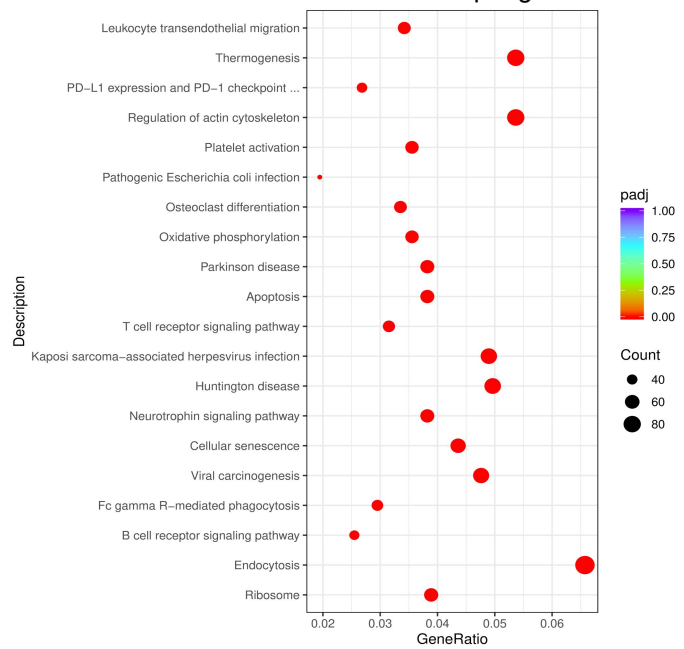

Supplement: Supplementary file 1 [file animals-15-00752-s001.zip › Supplementary File/Figure S4.pdf]
